# Temozolomide- and fotemustine-induced apoptosis in human malignant melanoma cells: response related to MGMT, MMR, DSBs, and p53

**DOI:** 10.1038/sj.bjc.6604856

**Published:** 2009-01-06

**Authors:** S C Naumann, W P Roos, E Jöst, C Belohlavek, V Lennerz, C W Schmidt, M Christmann, B Kaina

**Affiliations:** 1 Department of Toxicology, University of Mainz, Mainz, Germany; 2 Third Department of Internal Medicine, University of Mainz, Mainz, Germany; 3 Queensland Institute of Medical Research, Queensland, Australia

**Keywords:** temozolomide, fotemustine, melanoma therapy, apoptosis, MGMT, mismatch repair

## Abstract

Malignant melanomas are highly resistant to chemotherapy. First-line chemotherapeutics used in melanoma therapy are the methylating agents dacarbazine (DTIC) and temozolomide (TMZ) and the chloroethylating agents BCNU and fotemustine. Here, we determined the mode of cell death in 11 melanoma cell lines upon exposure to TMZ and fotemustine. We show for the first time that TMZ induces apoptosis in melanoma cells, using therapeutic doses. For both TMZ and fotemustine apoptosis is the dominant mode of cell death. The contribution of necrosis to total cell death varied between 10 and 40%. The O^6^-methylguanine-DNA methyltransferase (MGMT) activity in the cell lines was between 0 and 1100 fmol mg^−1^ protein, and there was a correlation between MGMT activity and the level of resistance to TMZ and fotemustine. MGMT inactivation by O^6^-benzylguanine sensitized all melanoma cell lines expressing MGMT to TMZ and fotemustine-induced apoptosis, and MGMT transfection attenuated the apoptotic response. This supports that O^6^-alkylguanines are critical lesions involved in the initiation of programmed melanoma cell death. One of the cell lines (MZ7), derived from a patient subjected to DTIC therapy, exhibited a high level of resistance to TMZ without expressing MGMT. This was related to an impaired expression of MSH2 and MSH6. The cells were not cross-resistant to fotemustine. Although these data indicate that methylating drug resistance of melanoma cells can be acquired by down-regulation of mismatch repair, a correlation between MSH2 and MSH6 expression in the different lines and TMZ sensitivity was not found. Apoptosis in melanoma cells induced by TMZ and fotemustine was accompanied by double-strand break (DSB) formation (as determined by H2AX phosphorylation) and caspase-3 and -7 activation as well as PARP cleavage. For TMZ, DSBs correlated significantly with the apoptotic response, whereas for fotemustine a correlation was not found. Melanoma lines expressing p53 wild-type were more resistant to TMZ and fotemustine than p53 mutant melanoma lines, which is in marked contrast to previous data reported for glioma cells treated with TMZ. Overall, the findings are in line with the model that in melanoma cells TMZ-induced O^6^-methylguanine triggers the apoptotic (and necrotic) pathway through DSBs, whereas for chloroethylating agents apoptosis is triggered in a more complex manner.

Melanoma is among the top six cancers responsible for cancer-related mortality worldwide (Balch *et al*, 2001). Once melanoma metastasises treatment outcome is poor. Only a few chemotherapeutic agents have been shown to be active in the treatment of melanomas. The most often used drug is dacarbazine (DTIC), which needs metabolic activation to generate the active DNA-methylating carbenium ions inside the cell (Hill, 1975). Temozolomide (TMZ), which is a triazene derivative that needs no metabolic activation, also has activity in melanoma cells. It hydrolyses spontaneously in aqueous solution, giving rise to the active metabolite 5-(3,3-methyltriazen-1-yl)imidazole-4-carboxamide that further decomposes to yield DNA-methylating species (Newlands *et al*, 1997). The response rate after treatment with these methylating drugs is ∼20% (Newlands *et al*, 1992; Bleehen *et al*, 1995; Middleton *et al*, 2000). Temozolomide penetrates well the blood–brain barrier and, therefore, it is also used for the treatment of brain metastases of melanomas (Bael *et al*, 2008). It can be administered orally and is well tolerated.

An important mechanism of resistance to methylating agents is DNA repair mediated by the damage-reversal suicide enzyme O^6^-methylguanine-DNA methyltransferase (MGMT). It repairs the pre-toxic DNA lesion O^6^-methylguanine (O^6^MeG) by transfer of the methyl group from guanine to an own cysteine residue (Pegg, 2000). This causes, in an expression-dependent manner, resistance to methylating agents that produce O^6^MeG (Kaina *et al*, 1991). Similar to malignant gliomas, melanomas express, compared to other cancers, quite low levels of MGMT (Chen *et al*, 1992; Kaina *et al*, 2007), which might explain why melanomas respond to the methylating drugs DTIC and TMZ, but not to many other anticancer drugs. In view of the role of MGMT in the defence against O^6^MeG one might anticipate that MGMT determines the clinical outcome in melanoma therapy with methylating drugs. However, conflicting data have been reported. Thus, the pretreatment levels of MGMT in biopsies of cutaneous tumours were not related to the outcome (Middleton *et al*, 1998) whereas a later study showed that MGMT expression in melanoma metastases was related to the clinical response of the patients to DTIC-based therapy (Ma *et al*, 2002a, 2003). Conversely, MGMT promoter methylation was not clearly related to the patients response upon TMZ treatment, although methylation threshold levels were also discussed to have an impact on therapy (Rietschel *et al*, 2008). It appears that melanoma cells are intrinsically resistant and acquire drug resistance by different strategies (Soengas and Lowe, 2003) in which MGMT is only one of several players.

The O^6^-methylguanine-DNA methyltransferase also causes resistance to chloroethylating agents like BCNU and the nitrosourea analogue, fotemustine (for review see Kaina *et al*, 2007). These agents are used in the therapy of metastatic melanoma either as a single agent (Li and McClay, 2002) or in combination with methylating agents, notably TMZ (Tas *et al*, 2007). Therapy with fotemustine has response rates of ∼20%; only partial responses were observed with a median of several months. The poor outcome of metastatic melanomas to chemotherapy stresses the need for improved strategies. A prerequisite for this would be the detailed knowledge of how these drugs exert their cell killing effects in malignant melanoma cells.

Temozolomide methylates DNA forming, among other lesions, O^6^MeG. The mechanism of apoptotic cell death triggered by O^6^MeG has been studied extensively in different cell systems, including rodent cells (Tominaga *et al*, 1997; Meikrantz *et al*, 1998; Ochs and Kaina, 2000; Roos *et al*, 2007b), human lymphocytes (Dunkern *et al*, 2003; Hickman and Samson, 2004; Roos *et al*, 2004), and malignant glioma cells (Roos *et al*, 2007a). It has become clear that O^6^MeG mispaired with thymine is the critical secondary pre-apoptotic DNA lesion. It has been proposed that the processing of O^6^MeG/T mismatches by MutS*α*-dependent DNA mismatch repair (MMR) gives rise to activation of ATR-Chk1 signalling (Caporali *et al*, 2004; Stojic *et al*, 2004) and DNA double-strand breaks (DSBs) that act as the ultimate trigger of the apoptotic pathway (Ochs and Kaina, 2000; Roos and Kaina, 2006). Alternatively, MutS*α*/MutL*α* bound to O^6^MeG/T lesions may directly activate apoptotic signalling. Apart from the finding that ATR/ATRIP is activated in the presence of MutS*α*-bound O^6^MeG/T mismatches (Yoshioka *et al*, 2006), evidence for this model is scarce. Contrary to TMZ, fotemustine modifies DNA by chloroethylation, giving rise to O^6^-chloroethylguanine. This lesion undergoes spontaneous inter- and intra-molecular rearrangement to form a N1-guanine-N3-cytosine interstrand crosslink (ICL; Tong *et al*, 1982). Although several other adducts are also formed, these ICLs are believed to be responsible for the killing effect of chloroethylating agents. Because MGMT repairs O^6^-chloroethylguanine before the ICL can form, and O^6^MeG before the mismatch can form, the repair protein is considered as a key mechanism of resistance of tumour cells against fotemustine, TMZ, and other anticancer drugs with similar properties.

Although methylating and chloroethylating drugs are crucial in primary therapy of metastatic melanomas, it is not known how they exert killing effects in melanoma cells. In a recent study it was reported that TMZ does not induce apoptosis, but senescence in human melanoma cells (Mhaidat *et al*, 2007), which is surprising in view of the fact that other cell systems such as malignant gliomas undergo apoptosis very efficiently in response to TMZ (Roos *et al*, 2007a). For fotemustine, it has been shown that melanoma cells are killed by apoptosis (Passagne *et al*, 2003; Kissel *et al*, 2006), but detailed studies on the mode of cell death by this drug in melanoma cells are not available. Also, there is no systematic study (utilising the same cell model) for melanomas as to the role of MGMT, p53, and MMR proteins in the O^6^-alkylguanine-triggered killing response. Here, we investigated the mode of cell death induced by TMZ and fotemustine in a panel of human cutaneous malignant melanoma cell lines. Notably, we assessed the question of cell death pathways (apoptosis, necrosis) evoked by these drugs in melanomas. We provide, for the first time, evidence that TMZ is a potent inducer of apoptosis in melanoma cells. We also show that fotemustine has the ability to induce both apoptosis and necrosis at high level. We furthermore demonstrate the formation of DSBs (*γ*H2AX) and caspase activation, and elucidated the impact of MGMT, MMR, and p53 status on the response.

## Material and methods

### Cell culture and drug treatment

The cell lines D03, D05, D14, MeWo, MeWo Fote40, MZ7, and SK29 were grown in RPMI-1640 medium containing 10% fetal calf serum, G361 was grown in McCoy's 5A medium containing 10% fetal calf serum, A375 and RPMI7951 were grown in DMEM containing 10% fetal calf serum, and Malme 3M was grown in DMEM containing 20% fetal calf serum. All cell lines were grown at 7% CO_2_, 37°C in medium containing 100 U ml^−1^ penicillin and 100 *μ*g ml^−1^ streptomycin. SK29-MEL (Wolfel *et al*, 1995), MZ7-MEL cells (Lennerz *et al*, 2005), D03-, D05-, and D14-MEL (Pavey *et al*, 2004; Stark and Hayward, 2007), and A365, Malme 3M, G361, and RPMI7951 (Haapajarvi *et al*, 1999) cells were described previously. *N*-methyl-*N*′-nitro-*N*-nitrosoguanidine (MNNG; Sigma, Munich, Germany) stocks were prepared by dissolving the drug in dimethyl sulphoxide (DMSO) and then diluting with sterile dH_2_O to a 10 mM concentration. Temozolomide (Schering-Plough, Kenilworth, NJ, USA) stocks, with 35 mM end concentration, were prepared by dissolving the drug in pure DMSO. The MNNG and TMZ stocks were filtered, aliquoted, and then stored at −80°C till use. Fotemustine (diethyl1-[3-(2-chloroethyl)-3-nitrosoureido]ethylphosphate; Muphoran, Servier Research International, Neuilly sur Seine, France) was prepared fresh for each treatment in a concentration of 1 *μ*g ml^−1^ in one-third of ethanol and two-thirds sterile distilled H_2_O. To deactivate the MGMT protein, 10 *μ*M O^6^-benzylguanine (O^6^BG) was added to the cells 1 h before drug treatment.

### Determination of apoptosis

*Sub-G_1_* Non-adherent and trypsinised adherent cells were combined, suspended in cold PBS, and fixed in ice-cold 70% ethanol for a minimum of 30 min. DNA in the cells was stained with propidium iodide (PI; 16.5 *μ*g ml^−1^) in PBS after RNase (0.03 *μ*g ml^−1^) digestion. For each sample 10 000 cells were subjected to flow cytometric analysis using an FACS Calibur (Becton Dickinson, Heidelberg, Germany). The number of apoptotic cells was calculated using the computer program WinMDI 2.8 (Joseph Trotter, http://facs.scripps.edu/software.html).

*Annexin V/PI* This assay distinguishes between early apoptotic cells and late apoptotic/necrotic cells, by using annexin V/PI double staining of unfixed cells. Cells in the supernatant and trypsinised cells were combined and suspended in 50 *μ*l binding buffer (10 mM HEPES (pH 7.4), 140 mM NaCl, 2.5 mM CaCl, 0.1% BSA). Annexin V-FITC (2.5 *μ*l; BD Pharmingen, Heidelberg, Germany) was added to each samples. After 15 min incubation in the dark, 430 *μ*l binding buffer and 1 *μ*g ml^−1^ PI per sample were added. The flow cytometric analysis was carried out using an FACS Calibur (Becton Dickinson). For each sample 10 000 cells were analysed. The evaluation of the cell populations was performed using the computer program WinMDI 2.8 (Joseph Trotter).

### Preparation of protein extracts

For preparing whole-cell extracts, cells were washed in ice-cold PBS, harvested, and re-suspended in whole-cell extract buffer (20 mM Tris-HCl; pH 8.5, 1 mM EDTA, 1 *μ*M *β*-mercaptoethanol, 5% glycerol, 10 mM DDT, 0.5 mM PMSF, 1 mM Na_3_VO_4_, proteinase inhibitor Complete; Roche, Mannheim, Germany). After sonication on ice (two times for 10 pulses) with a Branson sonifier (Cell Disruptor B15), output control=4, 40% Duty Cycle, the homogenates were centrifuged (10 000 **g**, 10 min at 4°C), and the clear supernatants were stored at −80°C.

For fractionated extracts, cells were washed in ice-cold PBS, harvested, and re-suspended in fractionation buffer A (10 mM HEPES-KOH; pH 7.4, 0.1 mM EDTA, 1 mM EGTA, 250 mM sucrose, 10 mM DDT, 0.5 mM PMSF, 1 mM Na_3_VO_4_, proteinase inhibitor Complete; Roche). Cell membranes were disrupted by freezing in liquid nitrogen and thawing at 37°C, four times (after thawing, the cell suspension was vortexed for 5 s). After centrifugation of the homogenates (700 **g**, 10 min at 4°C) to separate the nuclei in a pellet, the supernatant, including the cytoplasmic fraction, was transferred to a new reaction tube whereas the pellet was kept on ice. The supernatant was cleared by centrifugation (10 000 **g**, 10 min at 4°C) and the pure cytoplasmic fraction was stored at −80°C. The pellet containing the nuclei was re-suspended in whole-cell extract buffer (20 mM Tris-HCl; pH 8.5, 1 mM EDTA, 1 *μ*M *β*-mercaptoethanol, 5% glycerol, 10 mM DDT, 0.5 mM PMSF, 1 mM Na_3_VO_4_, proteinase inhibitor Complete; Roche) and sonicated on ice with a Branson sonifier (Cell Disruptor B15), output control=4, 40% Duty Cycle, two times for 10 pulses, the homogenates were centrifuged (10 000 **g**, 10 min at 4°C) and the supernatant containing the nuclear proteins was stored at −80°C.

### Protein concentration determination

Protein concentrations were determined using the Bradford method (Bradford, 1976). Bradford reagent (200 *μ*l; 0.01% G240 brilliant blue (Saba), 5% ethanol, 10% H_3_PO_4_, 85% dH_2_O) was added to 10 *μ*l of a 1 : 10 dilution of the protein extracts. Following 15 min incubation in the dark, the absorption was measured by photometry at 595 nm. The protein concentration was determined using a calibration curve with BSA protein, taken in parallel.

### Western blot analysis

The method used here is based on the method by Renart *et al* (1979). Samples of 25 *μ*g of protein extracts were separated on a 7.5 or 12% SDS-polyacrylamide gel. Separated protein were blotted onto a nitrocellulose transfer membrane (Schleicher and Schuell, Dassel, Germany) in a Bio-Rad blot cell for 3 h at 300 mA using buffer consisting of 25 mM Tris-HCl, 86 mM glycine, and 20% methanol. The membranes were blocked for 1 h at room temperature in 5% (wt/vol) milk powder in TBS (150 mM HCl, 20 mM Tris pH 7.6) containing 0.1% Tween 20 (TBS–Tween) and incubated overnight at 4°C with the primary antibody (1 : 200–1 : 1000 dilution) in 5% (wt/vol) milk powder or BSA in TBS–Tween. The membranes were washed three times for 10 min in TBS–Tween each, incubated for 1 h with a horseradish-peroxidase-coupled secondary antibody (dilution 1 : 4000) (Amersham Biosciences AB, Munich, Germany) in TBS–Tween and washed again three times for 10 min in TBS–Tween. For developing the membranes, a chemiluminescence detection system (Amersham Biosciences AB) was used. Western blots were quantified by densitometric scanning of the blots. The antibodies used were anti-PARP1 and anti-Erk2 (Santa Cruz Biotechnology, Heidelberg, Germany), anti-caspase-3 and anti-caspase-7 (Cell Signaling, New England Biolabs, Frankfurt, Germany), and anti-*γ*H2AX (Upstate, Millipore, Schwalbach, Germany).

### Determination of MGMT activity

The MGMT activity was determined as previously described (Preuss *et al*, 1995). It was expressed as fmol of [^3^H]-methyl transferred from radioactively labelled DNA to protein per mg of total cell extract protein.

### Mismatch repair assays

The MMR activity was determined as previously described (Christmann and Kaina, 2000). For gel retardation assays, 29-nucleotide oligomers with the general sequence 5′-GGGCTCGAGCTGCAGCTGCTAGTAGATCT-3′ were annealed to oligomers with the general sequence 5′-GGGAGATCTACTAGNAGCTGCAGCTCGAG-3′ (*n*=C or T) and labelled with [^32^P]dATP using polynucleotide kinase. After incubation with nuclear proteins DNA–protein complexes were separated on polyacrylamide gels.

### Statistics

Statistical analysis was performed using the computer-based program GraphPad Prism. For comparing differences between two populations the unpaired *t*-test was performed. For regression fits, the linear equation and the exponential decrease equation was used.

## Results

**Figure 1:**
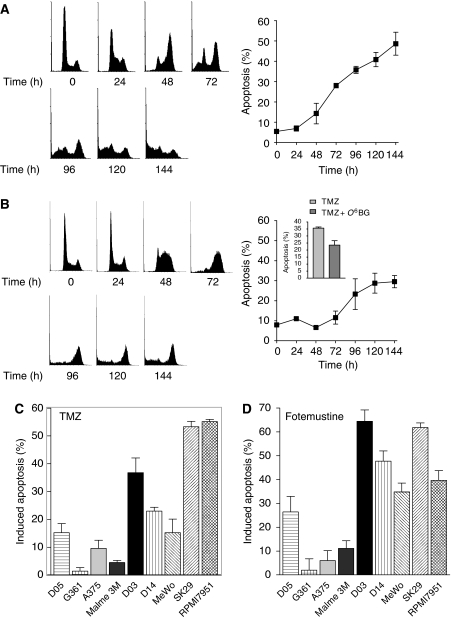
Apoptotic response after treatment with methylating agents in melanoma cells determined by quantifying the sub-G_1_ population using flow cytometry. (**A**, left panel) Time-dependent flow cytometry histograms after treatment of MeWo cells with 5 *μ*M MNNG, (**A**, right panel) time-dependent determination of apoptotic response following treatment of MeWo cells with 5 *μ*M MNNG. (**B**, left panel) Time-dependent flow cytometry histograms after treatment of MeWo cells with 50 *μ*M TMZ, (**B**, right panel) time-dependent determination of apoptotic response following treatment of MeWo cells with 50 *μ*M TMZ. (**C**) Frequency of apoptosis in nine melanoma cell lines after treatment with 50 *μ*M TMZ. Cells were pretreated with 10 *μ*M O^6^BG. (**D**) Apoptotic response in nine melanoma cell lines after treatment with 32 *μ*M fotemustine and 10 *μ*M O^6^BG. The baseline apoptosis level for the cell lines was as follows: D05, 5.3%; G361, 9.5%; A375, 2.5%; Malme 3M, 11.1%; D03, 15.5%; D14, 8.8%; MeWo, 6.7%; SK29, 5.2%; RPMI7951, 11.6%.

First, we studied the response of a well-known melanoma cell line, MeWo, to the model S_N_1-methylating agent MNNG. (This agent acts similar to TMZ and DTIC by inducing O^6^MeG adducts in the DNA.) As shown in Figure 1A , MNNG induces a time-dependent reduction in the fraction of G_1_ cells, an accumulation of cells in G_2_, and a dramatic increase in the level of apoptotic sub-G_1_ cells. This was most obvious late, that is 72–144 h, after treatment. Quantification of the sub-G_1_ population shows that apoptosis starts 2 days after MNNG treatment and increases with increasing post-exposure time (Figure 1A, right panel). This also holds true for TMZ that induced apoptosis 4 days after treatment (Figure 1B for representative histograms and time response of apoptosis of TMZ-treated MeWo cells). We should note that the dose of TMZ applied in these experiments (50 *μ*M) was rather low, that is below the maximal clinical dose level (optimal plasma peak level ∼100 *μ*M). Pretreatment of MeWo cells with O^6^BG did not lead to amelioration of TMZ-induced apoptosis (Figure 1B, inset of right panel). This is in line with the finding that MeWo cells do not express detectable MGMT (Christmann *et al*, 2001).

Next, we investigated a panel of melanoma cell lines that originated from cutaneous malignant melanomas. As depicted in Figure 1C, all but one cell line included in this study (D05, G361, A375, Malme 3M, D03, D14, MeWo, SK29, and RPMI7951) underwent apoptosis upon TMZ treatment, as measured by sub-G_1_. There were clear differences in the sensitivity of the cell lines, with RPMI7951 the most sensitive and G361 the most resistant. Similar experiments were performed with the chloroethylating drug fotemustine. As shown in Figure 1D, all but one melanoma cell line underwent apoptosis upon treatment, again with remarkably different sensitivities. D03 cells were the most sensitive and G361 cells were the most resistant. Interestingly, the onset of apoptosis was earlier for fotemustine- than for TMZ-treated cells; it was observed 24 h after the beginning of fotemustine treatment whereas for TMZ it was observed after 72–96h (Figure 1B and data not shown). It is important to note that all experiments with these cell lines were performed under O^6^BG pretreatment conditions to deplete residual MGMT activity.

**Figure 2:**
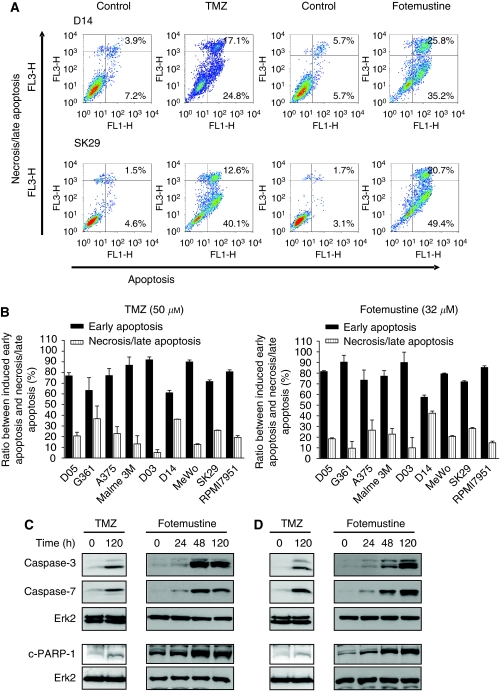
Quantification of the apoptotic and the necrotic/late apoptotic population by annexin V/PI double staining and western blot following TMZ and fotemustine treatment. (**A**) Representative flow cytometry density plots of cells not exposed and exposed to 50 *μ*M TMZ or 32 *μ*M fotemustine after 144 h in the presence of O^6^BG (10 *μ*M) in the melanoma cell lines D14 and SK29, (**B**) ratio between apoptosis and necrosis/late apoptosis in TMZ- or fotemustine-treated cells after 144 h treatment in eight cell lines. For Malme 3M the TMZ time point was 196 h. (**C**, **D**) Western blot analysis of effector caspase-3 activation documented by the cleavage products p17 and p19, effector caspase-7 activation documented by cleavage product p20 and cleavage of PARP-1 protein (c-PARP-1) following treatment with 100 *μ*M TMZ and O^6^BG (10 *μ*M), or 32 *μ*M fotemustine and O^6^BG (10 *μ*M) in two representative melanoma cell lines D14 (**C**) and SK29 (**D**).

The induction of apoptosis by TMZ and fotemustine in melanoma cells was confirmed using annexin V/PI double staining and flow cytometry analysis, which allows for simultaneous quantification of apoptosis and necrosis/late apoptosis (Vermes *et al*, 1995). Data shown in Figure 2A (representative density dot blots) and Figure 2B (for quantification) revealed that 60–90% of cell killing resulted from apoptosis. The D14 cell line displayed nearly the same amount of apoptosis (annexin-V-positive cells) and necrosis/late apoptosis (annexin-V- and PI-positive cells) both after treatment with TMZ and fotemustine. Necrosis was induced in the cell lines at a frequency between 10 and 40%.

Having shown that malignant melanoma cells are able to undergo apoptosis in response to TMZ and fotemustine, we studied the activation of caspases. We observed a clear activation of the executing caspases-3 and -7 upon TMZ and fotemustine treatment (Figure 2C for D14 cells and Figure 2D for SK29 cells). We also observed PARP cleavage, a hallmark of alkylation-induced apoptosis (Ochs and Kaina, 2000; Figure 2C and D). Overall, the data substantiate that malignant melanoma cells are able to undergo apoptosis after treatment with methylating and chloroethylating agents.

**Figure 3:**
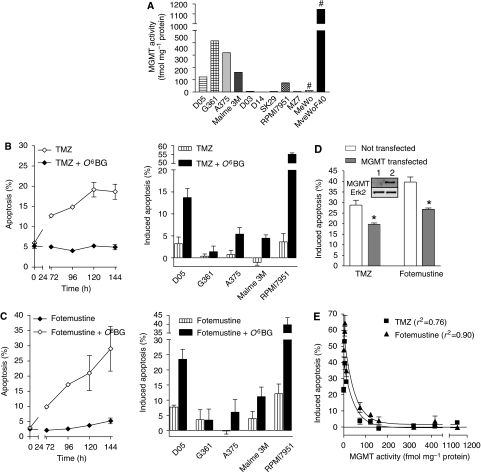
Determination of the influence of MGMT on alkylation at position O^6^ of guanine by treatment with TMZ and fotemustine. (**A**) Determination of MGMT activity (fmol mg^−1^ protein) in 11 melanoma cell lines. Time dependence of the apoptotic response as determined by quantifying the sub-G_1_ population by flow cytometry after treatment with 50 *μ*M TMZ with or without co-treatment with 10 *μ*M O^6^BG in MeWoF40 cells (**B**, left panel). Apoptotic response of D05, G361, A375, Malme 3M, and RPMI7951 cells to 50 *μ*M TMZ with or without co-treatment with O^6^BG 144 h later. For D05, A375, Malme 3M, and RPMI7951 the sensitisation was significant (*P*<0.05) (**B**, right panel). Time dependence of the apoptotic response as determined by quantifying the sub-G_1_ population by flow cytometry after treatment with 32 *μ*M fotemustine with or without co-treatment with 10 *μ*M O^6^BG in MeWoF40 cells (**C**, left panel). Apoptotic response of D05, G361, A375, Malme 3M, and RPMI7951 cells to 32 *μ*M fotemustine with or without co-treatment with O^6^BG 144 h later. For D05 and RPMI7951 the sensitisation was significant (*P*<0.05) (**C**, right panel). (**D**) Frequency of apoptosis as determined by quantifying the sub-G_1_ population by flow cytometry after treatment with TMZ and fotemustine for 120 h in non-transfected and MGMT transient transfected SK29 cells (^*^decrease of apoptotic response in MGMT transfected cells in comparison to control is significant, *P*<0.01). Expression of MGMT protein was determined by western blot analysis in non-transfected (lane 1) and MGMT transient transfected SK29 cells (lane 2) at the time of drug treatment. (**E**) Induced level of apoptosis after TMZ (50 *μ*M) or fotemustine treatment (32 *μ*M) as a function of MGMT activity level. Data points were fitted to a line depicting an exponential decrease with plateau at 0% induced apoptosis. The correlation is significant.

A major determinant of alkylating drug resistance is MGMT. Therefore, we determined the activity of this repair protein in the cell lines used in this study. They did not express detectable MGMT (D03, D14, SK29, MZ7, MeWo) or express MGMT at levels ranging between 100 and 400 fmol mg^−1^ protein (Figure 3A). For comparison, we included a MeWo-derived cell line MeWoF40 that was generated by continuous fotemustine selection (Christmann *et al*, 2001), which expressed MGMT at very high level (1160 fmol mg^−1^ protein; Figure 3A). MeWoF40 cells are highly resistant to TMZ. If MGMT was depleted by O^6^BG, apoptosis was induced at a high level, similar to MGMT-deficient MeWo cells (Figure 3B, left panel). Similar data were obtained with the cell lines D05, G361, A375, Malme 3M, and RPMI7951, all cell lines were MGMT positive and were sensitised following depletion of MGMT with O^6^BG; in four of the five lines (except G361 which is extremely resistant even under MGMT-depleted conditions) the sensitisation was significant (*P*<0.05; Figure 3B, right panel). This shows that O^6^MeG is the major apoptotic trigger in melanoma cells treated with TMZ.

MeWoF40 cells are also highly resistant to fotemustine. Under MGMT-depleted conditions, the cells approached the sensitivity of MeWo cells not expressing MGMT (Figure 3C, left panel) indicating that O^6^-chloroethylguanine, which is subject to repair by MGMT, is responsible for fotemustine-induced apoptosis in melanoma cells. This was confirmed with D05, G361, A375, Malme 3M, and RPMI7951 cells when MGMT was inactivated by O^6^BG (Figure 3C, right panel). All cell lines but one (G361, which was completely refractory to apoptosis) showed an increased sensitivity to fotemustine when MGMT was inhibited. To further show the protective effect of MGMT, SK29 cells were transiently transfected with MGMT cDNA. The data show that ectopic over-expression of MGMT significantly reduced apoptosis both after treatment with TMZ and fotemustine (Figure 3D). Further, we compared the apoptotic response with the MGMT level expressed in the cell lines utilised in the study. There was a significant correlation between the MGMT activity level and the degree of resistance to TMZ- and fotemustine-induced apoptosis (Figure 3E). This shows that in melanoma cell lines MGMT is a decisive marker of alkylating drug resistance.

**Figure 4:**
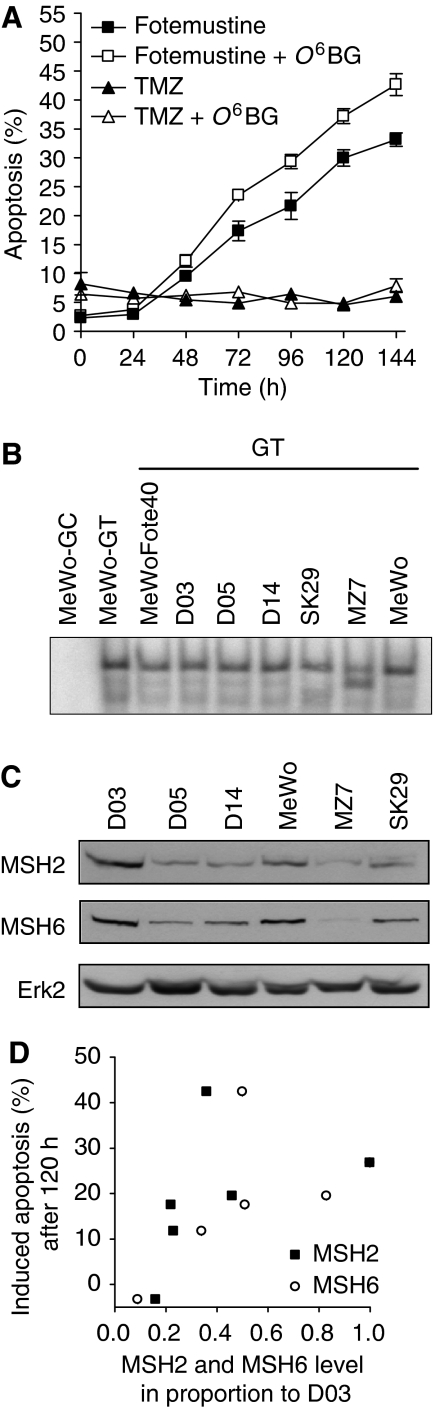
Influence of MMR on apoptotic response following TMZ or fotemustine treatment in the MMR-impaired cell line MZ7. (**A**) Time dependence of the apoptotic response as determined by quantifying the sub-G_1_ population by flow cytometry after treatment with 50 *μ*M TMZ or 32 *μ*M fotemustine with or without co-treatment with 10 *μ*M O^6^BG. (**B**) Measurement of the G/T binding activity of the MutS*α* complex in seven melanoma cell lines using the mismatch repair assay, (**C**) level of expression of MSH2 and MSH6 protein in different melanoma cell lines; Erk2 was used as loading control. (**D**) Induced level of apoptosis after TMZ treatment (50 *μ*M) as a function of MSH2 and MSH6 expression level (relative to D03 that was set to 1). The correlation is not significant.

Comparing the apoptotic response of different melanoma cell lines, one cell line (designated MZ7) was highly resistant to TMZ and did not undergo apoptosis (Figure 4A, left panel for a time-course experiment), although these cells did not express MGMT (Figure 3A). As expected, O^6^BG did not affect the response of these cells to TMZ (Figure 4A). When MZ7 cells were treated with fotemustine, they underwent apoptosis at a high level and O^6^BG slightly sensitised the cells (Figure 4A). Obviously, MZ7 cells have the ability to undergo apoptosis after exposure to fotemustine, but not TMZ. O^6^-MeG-triggered apoptosis requires DNA MMR, which is not essential for apoptosis induced by chloroethylating drugs (Liu *et al*, 1996; Pepponi *et al*, 2003). Therefore, we speculated that MZ7 cells are impaired in MMR. Indeed, the level of MutS*α* binding to a G/T mismatch-containing oligonucleotide was clearly different in MZ7 cells compared to the other melanoma cell lines studied, showing a protein–DNA complex with a lower molecular weight (Figure 4B). Thus, it appears that in MZ7 cells MMR is impaired, causing them to be highly resistant to TMZ. MutS*α* is composed of MSH2 and MSH6, the expression of which is shown in Figure 4C. MZ7 cells express a very low amount of MSH2 and MSH6 (at the border level of detection), which supports the inference that these cells are MMR impaired. The expression level of MSH2 and MSH6 in the other lines is higher than in MZ7 cells and quite variable. Importantly, comparing the response of the different cell lines, there was no correlation between MSH2 and MSH6 expression level and the sensitivity to TMZ (Figure 4D).

**Figure 5:**
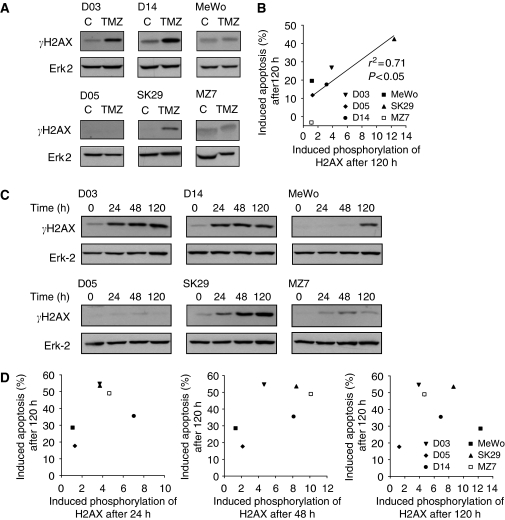
Phosphorylation status of H2AX in six melanoma cell lines. (**A**) Expression level of *γ*H2AX protein after treatment with 100 *μ*M TMZ and O^6^BG (10 *μ*M) for 120 h, (**B**) significant correlation (*P*=0.035) between induced H2AX phosphorylation and induced apoptotic response as determined by quantifying the sub-G_1_ population by flow cytometry 120 h after TMZ treatment (50 *μ*M). (**C**) Western blot analysis of *γ*H2AX after treatment with 32 *μ*M fotemustine and O^6^BG (10 *μ*M) for 24, 48, and120 h, (**D**) not significant correlation between induced *γ*H2AX phosphorylation for 24 h (left panel, *P*=0.354), 48 h (middle panel, *P*=0.154), and 120 h (right panel, *P*=0.885) and induced apoptotic response as determined by flow cytometry 120 h after fotemustine treatment.

On the basis of data obtained with repair-defective mutants, it was proposed that O^6^MeG lesions are converted by means of MMR and DNA replication into DSBs that act as a final trigger of apoptosis (Ochs and Kaina, 2000). Therefore, we were interested in determining the level of DSBs in response to TMZ and fotemustine in different melanoma cell lines. As an indicator of DSBs we measured the total amount of phosphorylation of histone H2AX, which occurs in response to DSBs (Rogakou *et al*, 1998). As shown in Figure 5A, *γ*H2AX was induced at different levels in the melanoma cell lines (Erk2 served as loading control). The highest level of induction was seen in D14, D03, and SK29 cells, the lowest level in MeWo, D05, and MZ7. Comparing the *γ*H2AX induction level with the apoptotic response, a significant correlation was found between both end points (Figure 5B), which supports the hypothesis that DSBs are involved in TMZ-induced cell death.

A similar study was performed for fotemustine. Figure 5C outlines the time course of *γ*H2AX induction. Unlike TMZ (data not shown), fotemustine induced *γ*H2AX already after 24 h, which is shown for the most sensitive lines D03, D14, and SK29. Overall, high level of *γ*H2AX induction was found in D03, D14, and SK29 cells, and low level of induction in MeWo, D05, and MZ7. Interestingly, the level of *γ*H2AX induction did not correlate at any of the time points (24, 48, and 120 h) with the level of fotemustine-induced apoptosis (Figure 5D). Thus, it appears that fotemustine-induced cell killing is based on a more complex mechanism in which DSBs may only be one of the several downstream components.

**Figure 6:**
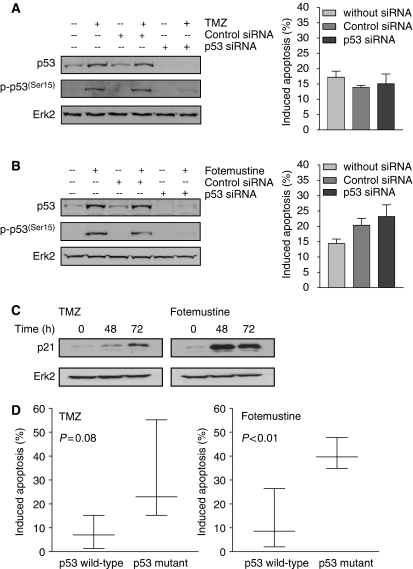
Activation of p53 and down-regulation by p53 siRNA. (**A**) D05 melanoma cells pretreated with O^6^BG (10 *μ*M) were treated with TMZ (50 *μ*M) and (**B**) fotemustine (32 *μ*M) and cells were harvested 72 h later. Control and p53 siRNA was transfected 24 h before the treatment with the drugs occurred. p53, p53 (Ser15 phosphorylated), and p21 were determined by western blots, and apoptosis (right panels) was quantified by sub-G_1_. Data are the mean of four independent experiments ±s.d. (**C**) Western blot analysis of the expression of p21 following treatment of cells with 50 *μ*M TMZ or 32 *μ*M fotemustine. ERK2 was used as loading control. (**D**) Apoptosis induced in melanoma cells that are wild type or mutated for p53 144 h following TMZ (50 *μ*M) (left panel) or fotemustine (32 *μ*M) (right panel). Data for the cell lines D05, G361, A375, Malme 3M, D14, MeWo, and RPMI7951 for which the p53 status is known were taken from Figure 1C and D.

In previous studies we showed that p53 determines the sensitivity of glioma cells to TMZ and ACNU (Batista *et al*, 2007; Roos *et al*, 2007a). Therefore, we hypothesised that p53 may also be involved in alkylating drug resistance in melanoma cells. D05 cells are wild type for p53 (Packer *et al*, 2007); it becomes stabilised upon treatment with TMZ (Figure 6A) and fotemustine (Figure 6B), which is in line with DSBs formed in response to the treatments. Stabilisation of p53 provoked up-regulation of p21 as observed 48 and 72 h after TMZ and fotemustine treatment (Figure 6C), which indicates that p53 is functional. Transfection of p53 siRNA nearly completely abolished basal p53 expression and prevented TMZ- and fotemustine-induced p53 up-regulation as well as p53 (Ser15) phosphorylation (Figure 6A and B). However, down-regulation of p53 by siRNA transfection did not impact the level of apoptosis after TMZ treatment (Figure 6A, right panel), indicating p53 does not stimulate TMZ-induced cell death. It slightly enhanced the level of apoptosis after fotemustine (Figure 6B, right panel), which is in line with a protective role of p53 for chloroethylating agents by stimulation of DNA repair (Batista *et al*, 2007).

If p53 does not stimulate TMZ-induced apoptosis in melanoma cells one would expect p53 wild-type melanoma cells not to be more sensitive than p53 mutant cells. This appears to be the case: in Figure 6D we compared p53 wild-type and p53 mutant melanoma cells. The comparison revealed that the cells expressing p53 wild type are, on average, more resistant to TMZ than p53-mutated cells (Figure 6D, left panel). p53 wild-type cells were also more resistant to fotemustine than lines expressing mutant p53 (right panel). As discussed below, the TMZ response is in marked contrast to the response of glioma cells.

## Discussion

First-line therapy of metastatic melanoma relies on the methylating and chloroethylating anticancer drugs DTIC, TMZ (Temodal), BCNU (Carmustine), and fotemustine (Muphoran). The mechanism of action of these agents at the DNA level has been well described. Temozolomide and DTIC methylate DNA at 13 sites (Kaina *et al*, 2007). One of the methylation products is O^6^MeG that, upon mispairing with thymine, activates MutS*α*-dependent MMR that triggers apoptosis (Kaina *et al*, 1997; Tominaga *et al*, 1997; Meikrantz *et al*, 1998). Fotemustine and other chloroethylating nitrosoureas induce O^6^-chloroethylguanine, which gives rise to N1-guanine-N3-cytosine crosslinks by inter- and intra-molecular rearrangement ∼8 h after induction of the primary lesion (Brent *et al*, 1987). The molecular cell death pathways triggered by the critical DNA lesions are not predictable and appear to be dependent on the cellular background. Thus, the same DNA lesion can induce apoptosis, necrosis, or cellular senescence and growth arrest (Roos and Kaina, 2006), the reason for which is often unclear. For TMZ, there is evidence that DSBs are involved as a downstream trigger of apoptosis (Roos *et al*, 2009), which is yet unknown for fotemustine. It has also been shown that p53 has a critical dual function. In gliomas, p53 wild-type cells are more sensitive to TMZ, but more resistant to ACNU than p53 mutant cells, which was explained by up-regulation of CD95 death receptor or DNA repair (Batista *et al*, 2007; Roos *et al*, 2007a).

In this study, we elucidated the mechanism of death of malignant melanoma cells upon treatment with TMZ or fotemustine. We show that melanoma cells treated with the agents die by apoptosis and necrosis, with apoptosis being the major death pathway. Apoptosis was accompanied by caspase-7 and -3 activation and PARP cleavage. This is in contrast to a recent report stating that TMZ induces senescence, but not apoptosis in melanoma cells (Mhaidat *et al*, 2007). The failure to observe apoptosis in that study might be explained by the different post-exposure times that were used. Thus, in the study by Mhaidat *et al* (2007) the expression of apoptosis marker such as caspase-3 and the cleaved PARP fragment was analysed 24–72 h after TMZ treatment. This seems to be a too short time period for apoptosis activation because TMZ-induced apoptosis is a late response that needs the passage of cells through at least two cell cycles after treatment (Kaina *et al*, 2007). Taking into account additional cell-cycle inhibition due to high-dose treatment, apoptosis in some cell types such as gliomas will only be visible 4–6 days after treatment (Roos *et al*, 2007a). Using time-course experiments we found that melanoma cells start undergoing apoptosis 72 h after the addition of TMZ to the medium. Interestingly, fotemustine induced apoptosis at earlier times, starting 24 h after treatment. This is to be expected because the toxic effect of chloroethylating agents does not rely on MMR (Liu *et al*, 1996; Pepponi *et al*, 2003) and, therefore, cytotoxicity is exerted earlier, likely in the treatment cell cycle. This has been recently shown for the cyclophosphamide analogue mafosfamide that induces, similar to fotemustine, ICLs that trigger apoptosis in S and G_2_ phases of the treatment cell cycle (Goldstein *et al*, 2008).

For TMZ, we show that *γ*H2AX is induced before apoptosis, which is indicative of the formation of DSBs. The frequency of DSBs was quantitatively correlated with the frequency of apoptosis. This supports a model where MMR generates gaps in the DNA (Mojas *et al*, 2007) that block replication, which leads to DSBs at collapsed replication forks in the second cell cycle after treatment (Ochs and Kaina, 2000; for review see Kaina *et al*, 2007). Thus, DSBs are postulated to act as a downstream trigger of O^6^MeG-induced apoptosis. The quantitative correlation between *γ*H2AX and sensitivity of melanoma cells to TMZ supports this notion and suggests that *γ*H2AX might be a useful predictive indicator for the melanoma response to a methylating-agent-based therapy. Interestingly, for fotemustine, a correlation between DSBs and the apoptotic response was not found, which indicates that for chloroethylating agents DSBs are not (or not the only) downstream apoptosis-triggering lesion. Contrary to O^6^MeG, ICLs prevent genes from being transcribed and, therefore, transcriptional inhibition could be involved. This conforms to previous findings with crosslinking agents for which transcriptional inhibition was shown to activate apoptosis (Ljungman *et al*, 1999; Arima *et al*, 2005; Goldstein *et al*, 2008). The question whether transcriptional inhibition has the main function in apoptosis triggered by therapeutic doses of fotemustine in melanoma cells is currently under study.

The cell death response of the melanoma lines was dependent on the MGMT level, which was shown by (1) the existence of an inverse correlation between the MGMT activity and TMZ-induced apoptosis, (2) inhibition of MGMT by *O*^6^-benzylguanine that sensitised all MGMT-expressing melanoma lines, and (3) transfection with MGMT which caused TMZ apoptotic resistance. The data are in line with other reports showing that MGMT protects *in vitro* against TMZ-induced melanoma cell death (Pepponi *et al*, 2003; Pagani *et al*, 2007). They are in line with the view that O^6^MeG and O^6^-chloroethylguanine are the most critical primary DNA lesions induced by TMZ and fotemustine in melanoma cells. Two cell lines (out of 11) were identified that exhibited a highly resistant phenotype. Band-shift experiments with extracts of one of these lines (MZ7) revealed an abnormal complex bound to a G/T oligonucleotide. Also, the expression of MSH2 was very low and MSH6 was not detectable indicating that this melanoma cell line is MMR defective. We should note that this line was generated from a malignant melanoma patient following treatment with DTIC. Therefore, the observed alteration in MMR might have occurred during therapy and might be an impressive example of acquired DTIC resistance. The extremely high level of resistance of another cell line, G361, to both TMZ and fotemustine is striking. It presumably relies on a downstream apoptosis execution defect, which is currently under study.

Abnormal levels of MMR proteins were reported in metastatic melanomas (Lage *et al*, 1999; Lo Muzio *et al*, 2000; Ma *et al*, 2002b; Shpitz *et al*, 2005). Because of this, and the high frequency of MMR-defective melanoma cell line (1 out of 11), down-regulation or impaired MMR might be considered (together with up-regulation of MGMT) a cause of acquired resistance to TMZ and other methylating drugs. This, however, does not explain the resistance of melanoma cells to fotemustine, with a low response rate similar to TMZ of ∼20% (Li and McClay, 2002). Therefore, it is possible that downstream defects, notably in the signalling and execution of apoptosis, are majorly responsible for melanoma drug resistance.

We also demonstrate activation of p53 upon TMZ and fotemustine treatment in melanoma cells. The apoptotic response of melanoma cells to TMZ was not related, however, to the p53 wild-type status. Thus, the cell lines expressing p53 wild type were, on average, more resistant to TMZ than p53 mutant cell lines (Figure 6D). Also, down-regulation of p53 by siRNA did not impact on TMZ sensitivity. This is in marked contrast to glioma cells that respond to methylating agents clearly better if p53 was wild type and worse if p53 was mutated or down-regulated (Roos *et al*, 2007a). For chloroethylating agents we found p53 wild-type melanoma cells again more resistant than p53 mutant cells (Figure 6D), which is in line with previous data obtained with glioma cells (Batista *et al*, 2007). Therefore, it appears that the role of p53 in the regulation of apoptosis upon TMZ treatment is different in melanoma cells than in gliomas and presumably other cancer cells. Whether and how this is related to the drug-resistant phenotype of melanoma cells is currently under study.
